# Biomechanical behavior of implant retained prostheses in the posterior maxilla using different materials: a finite element study

**DOI:** 10.1186/s12903-024-04142-8

**Published:** 2024-04-15

**Authors:** Ahmad Aboelfadl, Ludger Keilig, Kamal Ebeid, Mohamed Abdel Moniem Ahmed, Ingy Nouh, Ashraf Refaie, Christoph Bourauel

**Affiliations:** 1 Department of Fixed Prosthodontics, Faculty of Dentistry, Ain Shams University, Cairo, Egypt; 2 Oral Technology, Dental School, University Hospital Bonn, Bonn, Germany; 3 Department of Dental Prosthetics, Propaedeutics and Material Science, Dental School, University Hospital Bonn, Bonn, Germany; 4 Department of Fixed Prosthodontics, Faculty of Dentistry, Suez University, Suez, Egypt; 5 Department of Fixed Prosthodontics, Fayoum University, Fayoum, Egypt

**Keywords:** Biomechanics, Implant, Zirconia, PEKK, Von mises stress

## Abstract

**Background:**

The aim of this study is to evaluate the biomechanical behavior of the mesial and distal off-axial extensions of implant-retained prostheses in the posterior maxilla with different prosthetic materials using finite element analysis (FEA).

**Methods:**

Three dimensional (3D) finite element models with three implant configurations and prosthetic designs (fixed-fixed, mesial cantilever, and distal cantilever) were designed and modelled depending upon cone beam computed tomography (CBCT) images of an intact maxilla of an anonymous patient. Implant prostheses with two materials; Monolithic zirconia (Zr) and polyetherketoneketone (PEKK) were also modeled .The 3D modeling software Mimics Innovation Suite (Mimics 14.0 / 3-matic 7.01; Materialise, Leuven, Belgium) was used. All the models were imported into the FE package Marc/Mentat (ver. 2015; MSC Software, Los Angeles, Calif). Then, individual models were subjected to separate axial loads of 300 N. Von mises stress values were computed for the prostheses, implants, and bone under axial loading.

**Results:**

The highest von Mises stresses in implant (111.6 MPa) and bone (100.0 MPa) were recorded in distal cantilever model with PEKK material, while the lowest values in implant (48.9 MPa) and bone (19.6 MPa) were displayed in fixed fixed model with zirconia material. The distal cantilever model with zirconia material yielded the most elevated levels of von Mises stresses within the prosthesis (105 MPa), while the least stresses in prosthesis (35.4 MPa) were recorded in fixed fixed models with PEKK material.

**Conclusions:**

In the light of this study, the combination of fixed fixed implant prosthesis without cantilever using a rigid zirconia material exhibits better biomechanical behavior and stress distribution around bone and implants. As a prosthetic material, low elastic modulus PEKK transmitted more stress to implants and surrounding bone especially with distal cantilever.

## Background

Implant prosthetic frameworks generally maintain implant in their intended planned positions and regulate stress distribution on implants and surrounding bone [1, 2]. Occlusal stress concentration can cause harmful overloading on implant and overall peripheral bone structure leading to accelerated bone loss. This occurs because the response of bone cells, specifically in terms of bone formation and resorption, is influenced by local mechanical strains that occur during implant loading [3, 4]. 

Implants distribution together with prosthetic design highly affect the biomechanical behavior & long term implant success, wider implant distribution without a framework cantilever is likely to result in better biomechanical response and less technical complications in prosthetic components [5, 6]. However, due to limited availability of viable osseous tissue, bone density and proximity to vital structures, dental implants are commonly presented to the prosthodontist where cantilevers are unavoidable. The biological costs associated with reconstructive procedures are elevated owing to the need for bone grafting, harvesting procedures, and utilizing semipermeable barriers. In contrast, cantilever prostheses supported by implants facilitate more straightforward rehabilitation procedures and less surgical intervention with long-term success as reported in several studies [7–10]. 

Selection of prosthetic material is another critical clinical decision to guarantee even load distribution and long-term implant serviceability. Recently in modern implant dentistry, esthetic requirements, mechanical properties and peri implantitis prevention are considered highly critical perspectives for prosthetic material selection [11]. 

The rigidity in terms of modulus of elasticity is the most critical material property affecting the stresses generated in the implant prosthetic assembly. Modulus of elasticity varies among materials, being of high values in zirconia as a rigid material compared to more resilience in hybrid ceramics and polymers owing to their polymeric matrix [12]. Monolithic zirconia has been introduced to the profession with high esthetic properties and excellent antagonistic wear characteristics, yet maintaining high flexural strength, low fracture probability and surface roughness properties similar to conventional zirconia [13]. 

Polyetherketoneketone (PEKK) is a high-performance thermoplastic polymer originating from the PAEK (PolyArylEtherKetone) materials family claimed to provide superior mechanical properties owing to the double ketone bond in the chemical structure. The PEKK material based on the semi-crystalline structure, its resiliency and shock-absorbing capacity raise the possibility of using it as an implant prosthetic material [14, 15]. 

Among different implant prosthetic designs and materials, clinicians face uncertainty in determining the best prosthetic choice for effectively dissipating the generated stresses and ensuring long-lasting implant durability within the posterior maxilla. The primary criteria for making this selection focused on minimizing strain generation in the bone tissue and reducing stress within the implant/abutment prosthetic assembly to promote treatment longevity [16]. 

Finite element analysis (FEA) is a widely used valuable method for implant and surrounding bone stress analysis. The computational data generated from this analysis reliably show the biomechanical behavior and the effect of different designs, prosthetic components, connection types and recent materials on stress distribution under simulated clinical conditions [17, 18]. 

In literature, a diversity of FEA studies discussed the potential effect of different designs and cantilever extensions on stress distribution. Many authors reported detrimental effects of horizontal cantilever on implants and supporting structures, [19–21] while other limited studies concluded that increasing cantilever length did not cause distinct stress increase, however it might decrease stress values at distally tilted posterior implants and monolithic zirconia framework [22, 23]. 

The effect of implant prosthetic material has been similarly investigated by several FEA studies reporting controversial results regarding the effect of material properties especially the modulus of elasticity on stress distribution patterns [24–27]. Many studies reported direct relationship between the prosthetic material and its biomechanical effect on the prosthesis-implant assembly & surrounding bone [28–30], Conversely, other studies agreed that occlusal prosthetic materials did not interfere with peri-implant stress distribution [31–34]. 

Thus, this FEA study aims to evaluate the biomechanical performance of implant-retained prostheses in the posterior maxilla with different bridge designs comparing two prosthetic materials.

The first null hypothesis was that the biomechanical behavior of implant-retained prostheses is not affected by the bridge designs, while the second null hypothesis was that prosthetic materials would not influence the biomechanical stress distribution.

## Methods

### Geometric models

In this study, three identical FEA maxillary models (MF, MM and MD) were created, where two implants (tioLogic; Dentaurum, Germany) were placed in three different locations representing fixed fixed, mesial cantilever and distal cantilever implant prosthetic configurations respectively. In model (MF), two standard implants (tioLogic: 4.2 mm width, 7 mm length) were placed in the first premolar and first molar areas. In model (MM), the two implants were placed in the second premolar and first molar areas, while in model (MD), implants were placed in the first and second premolar areas. Implant prostheses with two materials; Monolithic zirconia (Zr) and polyetherketoneketone (PEKK) were modeled .The generated models were (MF-Zr, MM-Zr and MD-Zr) for zirconia material and (MF-PEKK, MM-PEKK and MD-PEKK) for polyetherketoneketone material.

### Modeling of Maxilla

A patient’s maxilla underwent scanning with a Planmeca ProMax 3D Mid-cone-beam computed tomography (CBCT) machine (Planmeca Inc., Helsinki, Finland) to generate an individualized FE model,. The scanning parameters were adjusted at 90 kV, a 12 mA X-ray beam current, and a 75 μm voxel dimension.

**Fig. 1:**
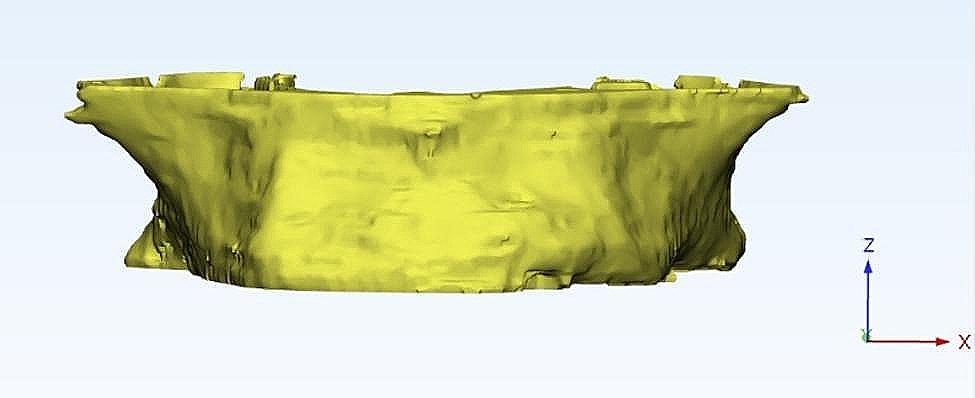
Individualized maxilla FE model based on anonymous patient CBCT

The acquired scan data were introduced into Mimics Innovation Suite, Mimics 14.0 / 3-matic 7.01; Materialise, Leuven, Belgium) to create a surface model without noise. This software was utilized for a 3D image processing and editing. Microscopic protrusions and holes were removed during CT image editing to minimize computational time (Fig. 1).

**Fig. 2:**
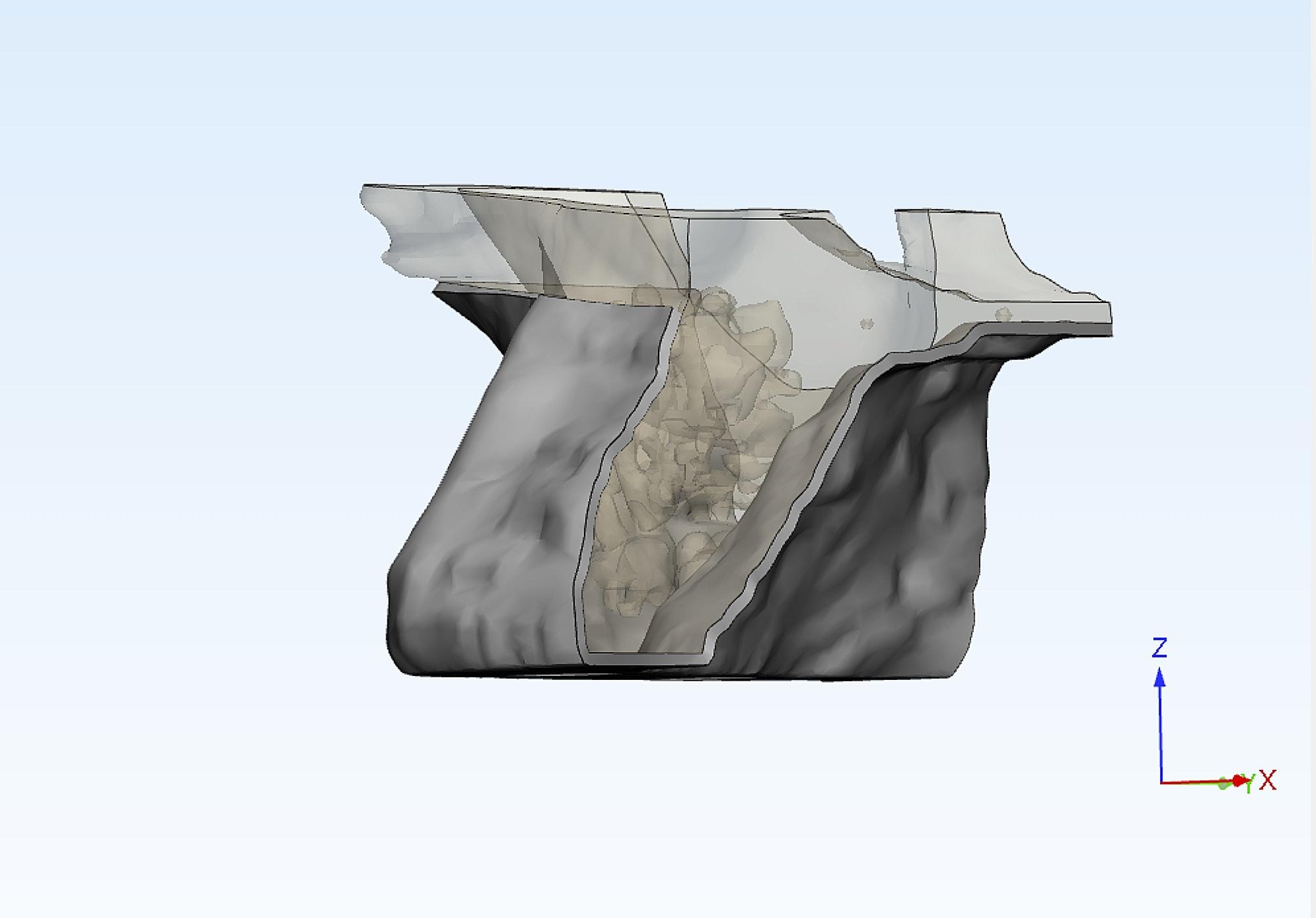
Compact and spongy bone masks created by Boolean operation

To simulate the clinical situation precisely, bone segmentation masks for compact and spongy bone structures were generated with region-growing and thresholding tools in Mimics. Afterward, the masks that were generated underwent separation utilizing Boolean operations, resulting in the display of both bone structures depicted in distinct colors (Fig. 2).

Subsequently, the process of obtaining the FE model involved the use of a computer-aided design (CAD) method using the triangulated surface mesh file format (STL) in Mimics. The 3D models were transferred into 3-Matic software, where they underwent a conversion process into STL files, ultimately generating the surface meshes. The outer surface of the segmented bone was used as a reference to fit an analytical surface. This analytical surface served as the foundation for designing a solid bone model, also known as a volumetric model.

### Implant prosthetic model generation

Creating an accurate analytical model involved modeling all potential factors that might have an impact within the specific region under investigation.

**Fig. 3:**
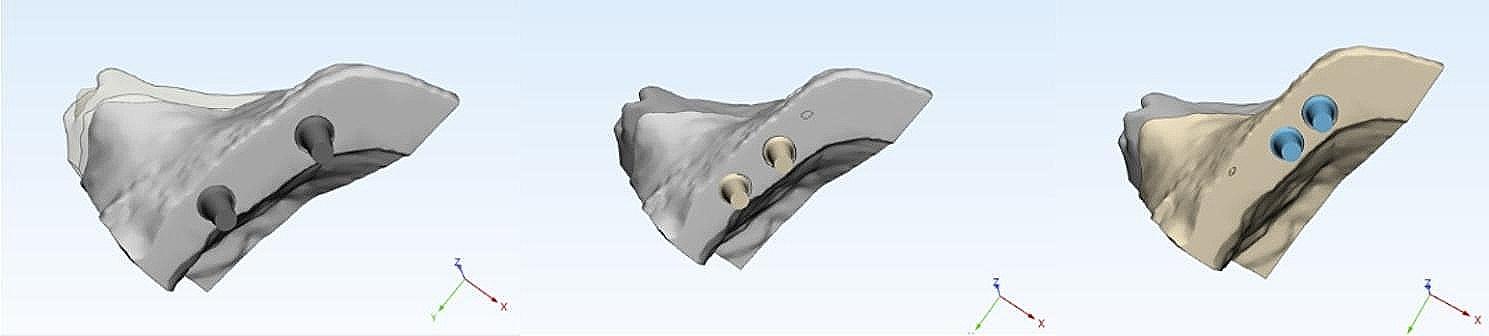
(**A**)Fixed-fixed ,(**B**) Mesial cantilever and (**C**) Distal cantilever designs

The STL data of each component, tioLogic Implant Fixture (4.2 mm width, 7 mm length) and abutments (tiologic anatomic abutment) were transferred into the 3D modeling software 3 Matic* (Materialise, Leuven, Belgium) for reducing the size of the STL files while maintaining their quality. The implant 3D solid model was duplicated and positioned at the center of the 3D bone model, 1 mm above the bone level, with three implant distributions to correspond to the three experimental models with the respective placements (MF, MM and MD) .(Fig. 3).

After obtaining the precise implant positions, the implant insertion holes in the solid bone model were obtained by Boolean subtraction. Next, each model received anatomical prosthetic solid abutments corresponding to implant positions, followed by defining a 30-microns thick cement gap on each abutment to clinically mimic a layer of dual-polymerized resin cement (Panavia F 2.0; Kuraray Medical Inc.) [27, 33]. 

**Fig. 4:**
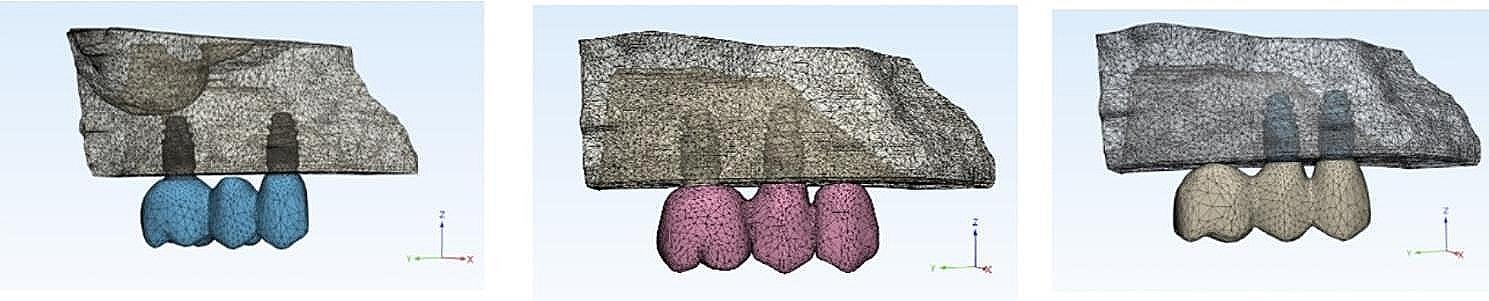
FE model of (**A**) Fixed fixed,(**B**) Mesial cantilever (**C**) Distal cantilever prostheses

Three implant prosthetic configurations were designed using the CAD method formulating fixed-fixed, mesial cantilever, and distal cantilever prostheses corresponding to the three experimental models. The job definition was an implant-retained full anatomic monolithic design with two prosthetic materials: monolithic zirconia (ZR) and polyetherketoneketone (PEKK). (Fig. 4), then the standard tessellation language (STL) files were transformed into solid models through meshing.

### Creation of mathematical models

The (STL) files of all generated solid models (MF-Zr, MM-Zr and MD-Zr) for zirconia material and (MF-PEKK, MM-PEKK and MD-PEKK) for polyetherketoneketone material were imported into Marc/Mentat (ver. 2015; MSC Software, Los Angeles, California) in the CDB file format for the FEA. At a singular integration point within each element, the stresses and strains were computed in every direction.

A 10-node tetrahedral element with six degrees of freedom at every node and a quadratic interpolation function was used for mesh generation. Using the tetrahedral element allows good adaption to irregular geometries and its quadratic interpolation function offers a more accurate alignment between real loading circumstances and non-linear behavior. The subdivision of.

the complex geometries into a finite number of elements was performed according to the mesh convergence test of 10% [35]. Thus, mesh was generated with the final models consisted of 316,109 elements and 87,023 nodes for the fixed-fixed model, 363,367 elements and 85,806 nodes for the mesial cantilever model, and 358,425 elements and 76,706 nodes for the distal cantilever model. (Table 1).

**Table 1:** Quantitative model information of the FE models

Nodes and Elements	Fixed-fixed model (MF)	Mesial cantilever model (MM)	Distal cantilever model (MD)
**Total of Nodes**	87,023	85,806	76,706
**Total of Elements**	316,109	363,367	358,425

### Interface conditions

It was assumed that all materials used in the study were homogeneous, isotropic, and exhibited linear elasticity. The bone-to-implant interface was presumed to be fully bonded with zero frictional contact to simulate 100% osseointegration [28, 36]. 

**Fig. 5:**
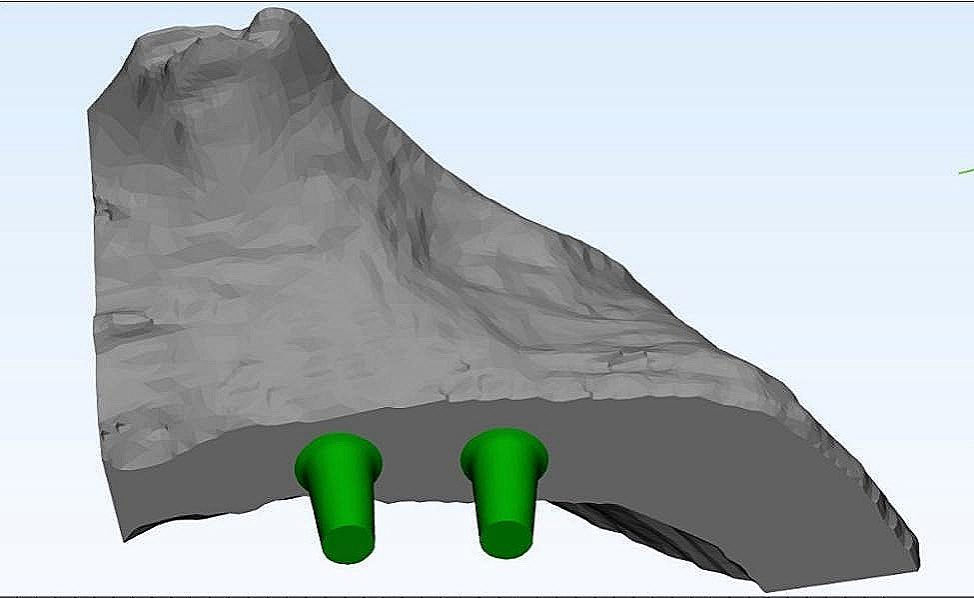
Abutments with virtual cement gap

The prosthesis-abutments assembly was cement-retained (virtual cement gap ) , where abutments are considered perfectly bonded to the prosthesis [19, 33]. (Fig. 5)

### Loading and boundary conditions

Loading and boundary parameters are prescribed for the FE models. Nonlinear geometric analyses were accomplished utilizing the commercial FE software Marc/Mentat (ver. 2015; MSC Software, Los Angeles, California). The mechanical attributes of elastic modulus and Poisson’s ratio for each material, as derived from prior published research, were inputted into the software (Table 2).

**Table 2:** Material characteristics of the FE models

Structure	Elastic modulus (GPa)	Poisson’s Ratio	References
**Bone**	13.7	0.30	Ramos Verri et al.,2015 [37]
**Implants**	110	0.34	Schwitalla et al., 2015 [38]
**Zr**	200	0.26	Al-Zordk et al., 2020 [39]
**PEKK**	3.5	0.36	Heimer et al., 2017 [40]

The boundary conditions restricted the movement and rotation of the base of the maxillary bone by fixing it in all three longitudinal directions as well as all three rotational directions. Similar restrictions were implemented into the bone ends, and to avoid distortion, one side of the bone area was also subjected to limitations due to its asymmetrical shape.

**Fig. 6:**
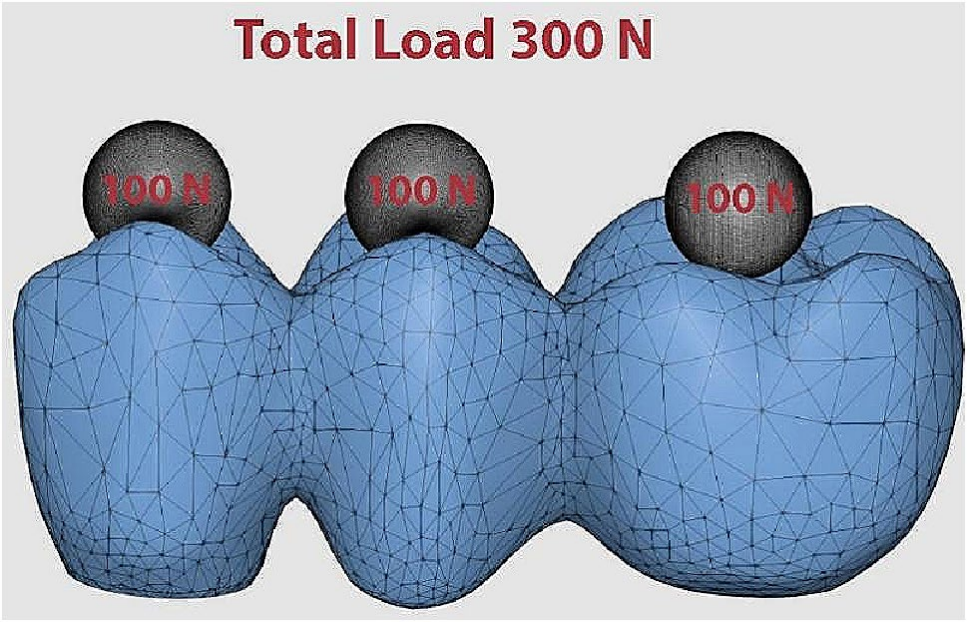
Axial load on experimental model

A 300 N load was statically applied in axial direction on each experimental model where each central fossa was subjected to 100 N load. (Fig. 6 )

Static analysis was accomplished, and the von Mises stresses for all models in the prosthesis, implant, and bone complex were calculated. Due to the limited sample size of one for each group in this finite element study, it was not feasible to conduct a comprehensive statistical analysis.

## Results

**Fig. 7:**
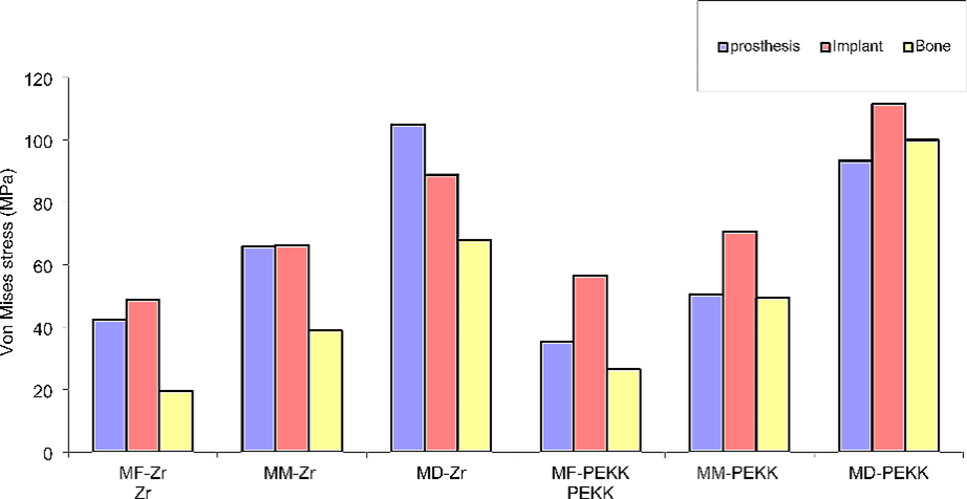
Von Mises stress in implants, prosthesis and bone descriptive charts

In the following, stresses in the prosthesis, implant, and bone are displayed in color coding, with red zones referring to high-stress values, while blue color gradients refer to low-stress values for each group. The maximum values were extracted from the graphs and listed in a descriptive chart (Fig. 7).

In general, the mesial and distal off-axial extensions generated much higher von Mises stresses than the fixed models. Finite stress peak (MPa) in prosthesis, implant, and compact bone under axial load in zirconia and PEKK models are presented in Table 3.

**Table 3:** Obtained values of finite stress peak (MPa) in prosthesis, implant and compact bone under axial load in zirconia and PEKK models

**Stress (MPa)**	Zirconia			PEKK		
	**Fixed** (MF-Zr)	**Mesial (**MM-Zr)	**Distal** (MD-Zr)	**Fixed** (MF-PEKK)	**Mesial (**MM-PEKK)	**Distal** (MD-PEKK)
**Prosthesis**	*42.4*	*65.9*	*105.0*	*35.4*	*50.6*	*93.3*
**Implant**	*48.9*	*66.3*	*88.9*	*56.6*	*70.7*	*111.6*
**Bone**	*19.6*	*38.9*	*67.8*	*26.5*	*49.4*	*100.0*

**Fig. 8:**
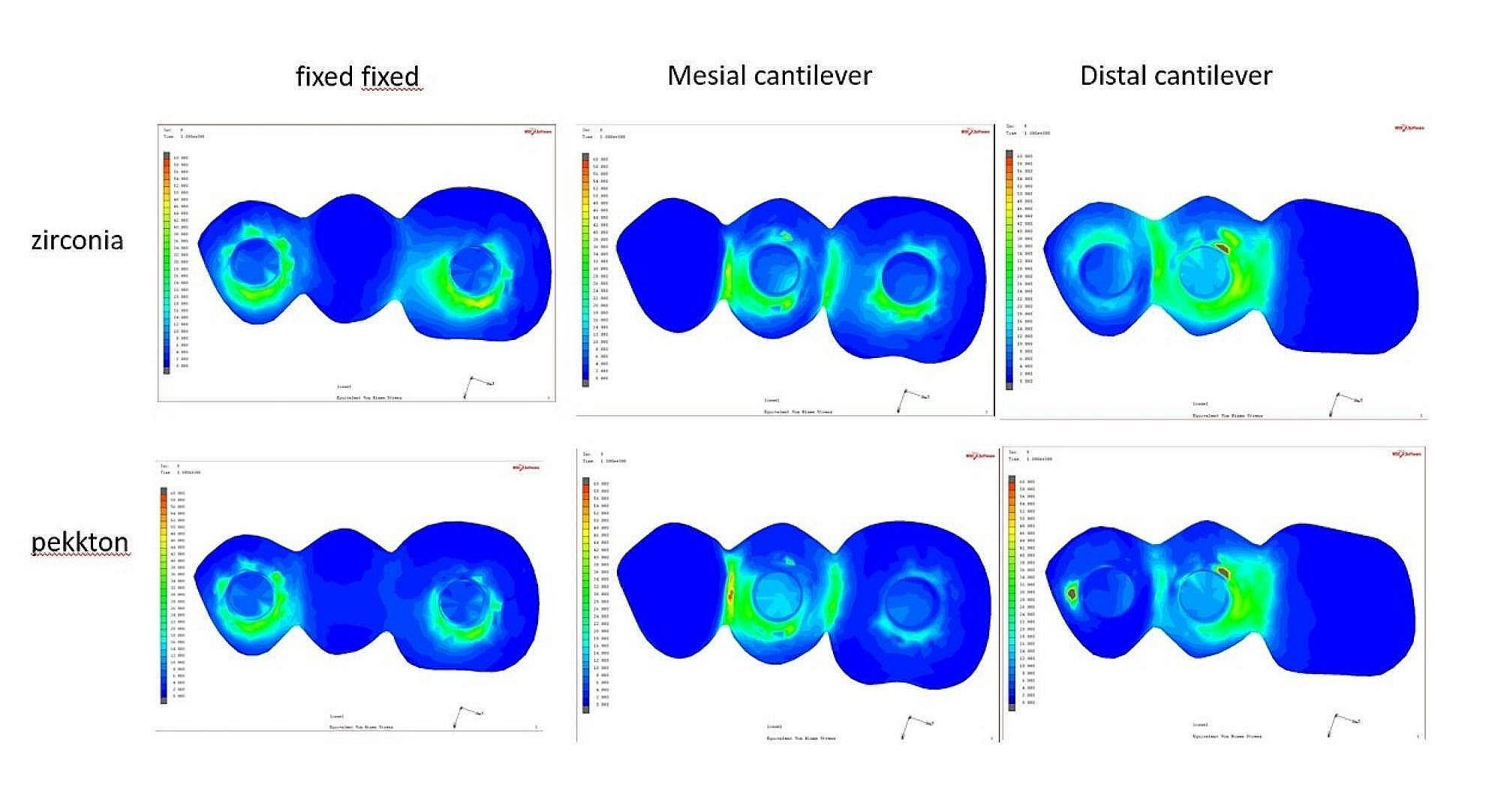
Distribution of Von Misses stress in the prosthesis. From above to below: zirconia and PEKK prostheses. From left to right, fixed-fixed, mesial cantilever, and distal cantilever designs

Regarding the prosthesis, MD-Zr model yielded the highest von Mises stresses of 105 MPa, with concentrated patterns at the abutment prosthesis interface adjacent to the offset extension, followed by the MD-PEKK model (93.3 MPa). The MF-PEKK model showed the lowest von Mises stress (35.4 MPa), with equally distributed stress patterns at the implant prosthesis interface (Fig. 8).

**Fig. 9:**
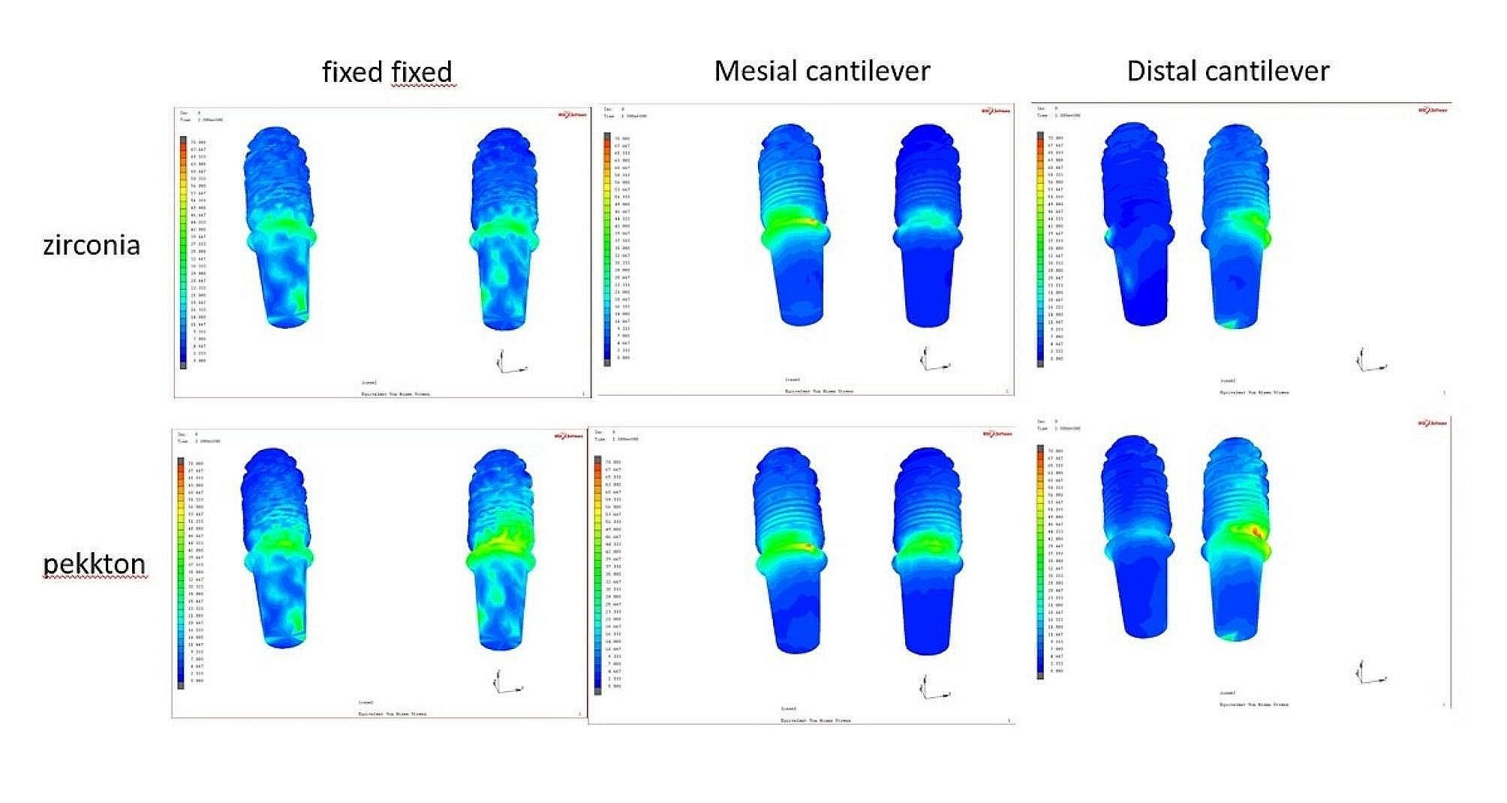
Distribution of Von Misses stress in implants. From above to below: zirconia and PEKK prostheses. From left to right, fixed-fixed, mesial cantilever, and distal cantilever designs

Regarding the stresses in implants, the maximum von Mises stresses (111.6 MPa) were generated in the MD-PEKK model, with concentrated stress adjacent to the distal offset extension. The lowest recorded von Mises stress scores (48.9 MPa) were observed in the MF-Zr model (Fig. 9).

**Fig. 10:**
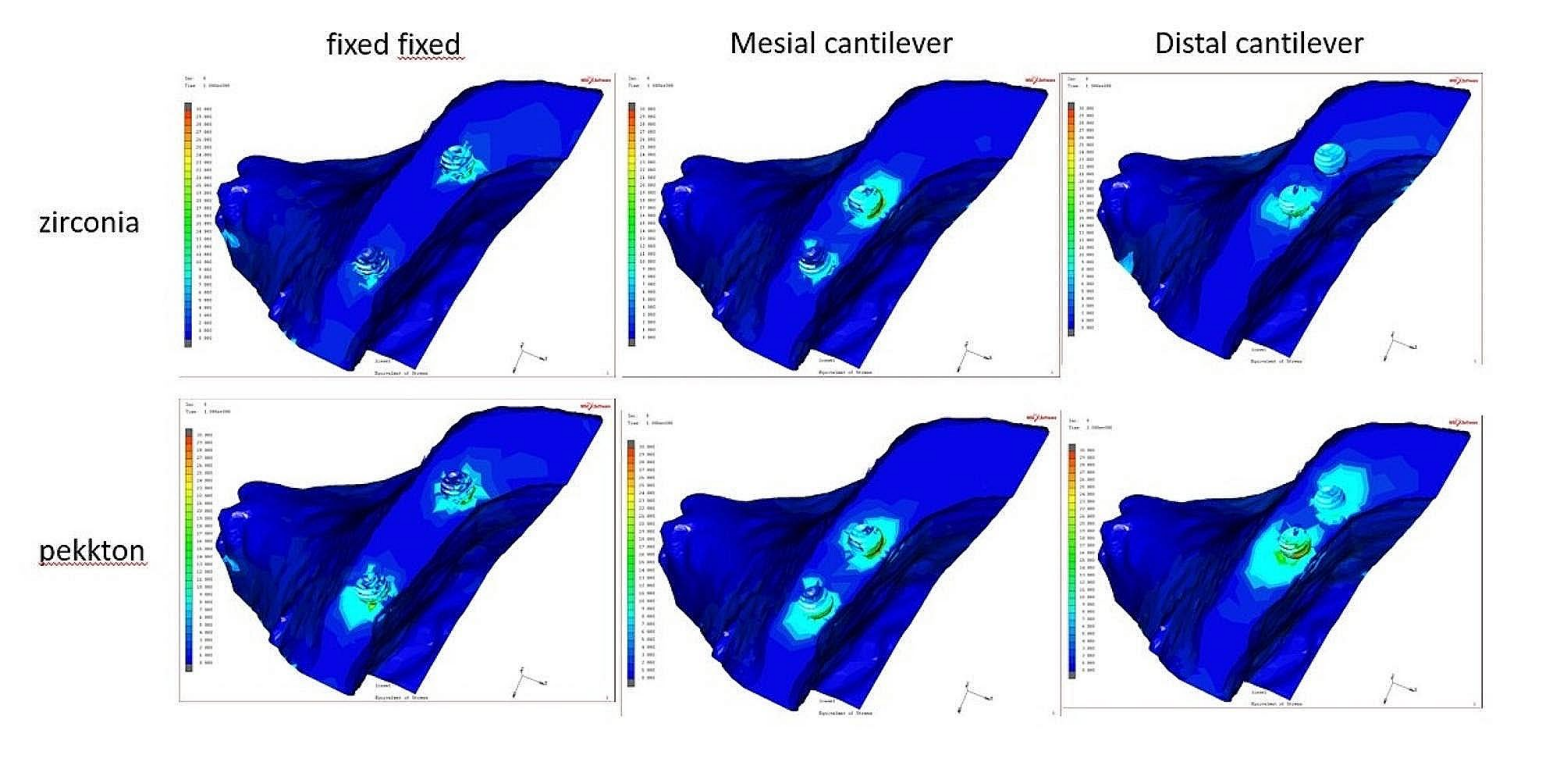
Distribution of Von Misses stress in bone. From above to below: zirconia and PEKK prostheses. From left to right, fixed-fixed, mesial cantilever, and distal cantilever designs

Regarding the induced von Mises stresses generated at the compact bone, the monolithic zirconia models showed lower von Mises stress than the PEKK models; the highest von Mises stresses (100.0 MPa )were presented in the MD-PEKK model, and the lowest values (19.6 MPa) were generated in the MF-Zr model with vertical loading (Fig. 10).

## Discussion

In the present study, the first null hypothesis was rejected as the results showed that the biomechanical behavior of implant-retained prosthesis was affected by the prosthetic bridge design, where the non-axial offset extensions generated higher total stresses compared to the fixed-fixed design.

Distal cantilever designs generally yielded the highest von Mises stresses in implants (111.6 MPa and 88.9 MPa ) in MD-PEKK and MD-Zr models respectively with concentrated stress adjacent to the distal offset extension, while fixed-fixed bridges without offset extensions showed the best biomechanical behavior with equally distributed stress patterns at the implant prosthetic interface and the lowest von Mises stress in implants(56.6 MPa and 48.9 MPa ) in MF-PEKK and MF-Zr models respectively. This might be attributed to the considerable bending moment, hinging effect together with rotational forces especially at the most distal areas [19]. 

These findings corroborate with many studies that reported the biomechanical risk of distal cantilever implant prostheses especially in patients with high masticatory overload or parafunctional habits [41–44]. Similarly, an in vitro study by Ahmed et al. evaluated the effect of different implant prosthetic designs on the biomechanical behavior using strain gauge analysis and concluded that the fixed-fixed bridge design is more advantageous in restoring the posterior edentulous area, particularly in terms of strain measurements on the prosthetic components, implant, and bone [45]. 

The results of our study have been compared for validity with other experimental studies in literature. One study was a finite element analysis similar to our work conducted by Ahmed et al. investigating the effect of prosthetic design and restorative material on the stress distribution of screw retained 3-unit implant-supported fixed partial dentures. Three FE models were virtually designed : fixed bridge (FB), cantilever bridge (CB), and separate crowns (SC) with two prosthetic materials : Multi-layered zirconia prothesis and a combination of PEEK (Polyetheretherketone) framework and multi-layered zirconia crowns. The results of this study reported better biomechanical behavior for fixed designs with PEEK framework and zirconia crowns. However, in this study screw retained prostheses were modelled and subjected to 100 N load rather than cement retained bridges that are subjected to 300 N load in the present study [46]. 

Based on the analysis of the current study,the highest von Mises stresses in bone (100.0 MPa and 67.8 MPa ) were recorded in distal cantilever models (MD-PEKK and MD-Zr respectively),while the lowest von Mises stress values were observed at the surrounding bone (26.5 MPa and 19.6 MPa) in models without cantilever extensions ( MF-PEKK and MF-Zr respectively ). These stress distribution patterns might be due to more favorable anteroposterior spread in models without cantilever as similarly observed by Doganay et al. who compared the stresses transmitted to implants with different implant inclinations with and without cantilever extensions, the study revealed that overall stress values were found to be higher in the cantilever model with tilted implants (von Mises:129 MPa). Moreover, they proved that distally placed implants, with consequent elimination of cantilever, decreased compressive stress values at the implants and the surrounding bone with the least values (–25 MPa) in the model with six vertical implants [21]. 

Comparebly, Yu et al. evaluated the biomechanical properties of different restoration configurations in implant-supported complete-arch fixed mandibular prostheses, this FEA study highlighted that avoiding posterior framework cantilever exhibited more favorable stress distribution conforming to the results of the present study [5]. Similar confirmative conclusions have been stated in a FEA study by Zhong et al. analyzing the biomechanical responses of implant supported monolithic zirconia fixed prosthesis with different implant configurations, where wider implants distribution avoiding cantilever extensions proved more balanced biomechanical performance with lower stress concentration on both implant and bone [6]. 

On the contrary, Ebadian et al. concluded that increasing cantilever length did not significantly raise stress values especially around the implant near to cantilever, however it is worth mentioning that this FEA study was conducted on mandibular implant supported overdentures rather than implant‑supported fixed prostheses as in the present study [22]. 

A systematic review on the biomechanics of implant-supported cantilevered fixed dental prosthesis suggested the use of cantilever designs (mesial or distal ) as a prosthetic alternative avoiding more invasive surgical procedures, yet the clinical criteria as cantilever length, occlusion scheme, implant number and diameter should be highly considered [47]. 

Likewise, another review article on implant-supported fixed prostheses with cantilever extensions concluded that they could be used as a reliable alternative treatment option in terms of long term implant survival rate and marginal bone level changes. Additionally, they stated that the decision of a distal or mesial cantilever depends on the clinical condition and mesial cantilevers are preferable due to more favourable force direction and better biomechanical lever forces around the implant acting as fulcrum ,yet other interrelated factors should be taken into consideration [48]. 

Those findings are consistent with our results regarding mesial cantilever, where the von mises stress in mesial cantilevers at implant interface were ( 66.3 MPa and 70.7 MPa ) in MM-Zr and MM-PEKK models respectively .These values are considerably less than the von mises stress ( 88.9 MPa and 116 MPa ) in distal cantilever models (MD-Zr and MD-PEKK respectively.) It should be also noted that increased surface area of first molar in distal cantilever in comparison to first premolar in the mesial cantilever might play a role in the more detrimental biomechanical effect of the distal cantilever designs .

In terms of implant prosthetic materials, the second null hypothesis was also rejected as the study results showed inter-relationship between the resultant stresses in the different structures and the elastic modulus of the prosthetic material. Regardless the prosthetic design, zirconia generally presented higher von Mises stresses in the prosthesis with the highest values (105.0 MPa ) in MD-Zr model, while PEKK generated the lowest stress values in the prosthesis (35.4 MPa) in MF-PEKK model .

These results are consistent with other studies suggesting that forces withstood by rigid materials as zirconia might be passed toward other parts of the implant restoration creating more stress dissipation around the cervical region of the abutment and abutment-implant junction .In this sense, a restorative material with high crystalline content as zirconia together with as stiff titanium substrate result in more favorable mechanical performance and better load bearing capacity compared to detrimental effects of resilient restorative materials due to the heterogenous passage of stresses between the elastic prosthetic material and more rigid titanium abutment [49, 50]. 

Regarding the effect of prosthetic material on stress distribution in implant and bone, the lowest values in implant (48.9 MPa) and bone (19.6 MPa ) were displayed in MF-Zr model and the highest von Mises stresses in implant (111.6 MPa) and bone (100.0 MPa) were recorded in MD-PEKK model with concentrated stress adjacent to the distal offset extension. These observations might highlight the biomechanical advantage of combining fixed fixed design with a rigid zirconia material on peri implant bone rather than the bending moment with detrimental effects of a combination of elastic polymer material with distal extension.

In the same context,our findings suggest that the material with lower elastic modulus as PEKK concentrated less stress on its prosthetic structure while inducing more stresses at the implant-bone interface. These findings are maintained by comparable ones in many studies concluding that shock absorbing polymeric frameworks of low elastic modulus induce more deformation and micromotion at the implant-bone interface transmitting more stresses to implants and surrounding bone from polymeric frameworks under occlusal loading, compared with the rigid materials that concentrate more stresses in the prosthesis itself than being transmitted to implant and bone [5, 30, 51, 52]. 

Similarly, a FEA study on implant supported over dentures reported more favorable stress distribution using standard rigid materials. In this study mandibular over dentures have been modeled with three framework materials (PEEK, Zantex, CoCr alloy) comparing their biomechanical behavior. The stresses with PEEK frameworks were observed at high values especially around the mini implants (11.24 N/mm2), while the least values (3,86 N/mm2) were displayed by CoCr around standard implants neck. A conclusion was drawn that using polymers as prosthetic framework materials exhibited unfavorable stress transmission compared to metal frameworks confirming more successful results with rigid materials [28]. 

In a FEA study by Yu et al., the authors compared different framework materials in mandibular full arch implant prosthesis with different implant configurations. The framework materials evaluated were pure titanium, cobalt-chromium alloy, type IV gold alloy, zirconia, polyetheretherketone (PEEK), and carbon fiberereinforced polyetheretherketone (CFR-PEEK).Based on their analysis, the rigid materials increased the framework stress values of approximately 9 to 103 MPa in all designs, compared with the polymeric material, while displaying less bone and peri implant stress compared with polymers that exhibited greater deformation and unfavorable stress concentration [5]. 

Güzelce et al. reported comparable results in a FEA study comparing different framework materials on maxillary palateless implant‑supported overdenture.Static loads of 150 N were applied to the first molar region of implant supported overdenture models with different framework materials (cobalt chromium alloy, glass, aramid, polyethylene, and carbon fiber-reinforced composites “FRC” ). The von Mises stresses on implants together with the maximum and minimum principle stresses on bone were analyzed. The highest von Mises stress values were observed in the aramid fiber supported models concentrating at implant neck (9.31 MPa) while the lowest values (8.97 MPa ) were presented in FRC supported models.Accordingly they recommended the clinical use of fiber materials with a high modulus of elasticity as an alternative to metal framework material [30]. 

In parallel with the previous study, same materials have been tested in a FEA study on fiber-reinforced maxillary overdentures, yet two different implant locations have also been examined and confirmative results were concluded. In addition, the study highlighted the advantageous stress levels and patterns in anteriorly placed implant location [24]. 

Conversely, in a FEA study by Mourya et al., PEEK material generated the least stresses in bone in comparison with porcelain fused to metal .They claimed that these results are due to the direct proportional tendency between the elastic modulus of the restorative material and the stress concentration between restoration/cement and cement/implant ,additionally they referred their results to the matching elastic modulus of PEEK material with bone that helps in creating a stress shielding effect preventing high stress peaks during load transfer at the bone-implant interface. However, this study was conducted on crowns over single implants rather than multiunit prosthesis and parafunctional rather than axial loading conditions have been applied [27]. 

In literature, other studies argued that restorative materials did not interfere with peri-implant stress distribution [16, 31–33]. Kaleli et al., analyzed six different models with different combinations of restoration materials (translucent zirconia [TZI], lithium disilicate glass ceramic [IPS], polymer-infiltrated hybrid ceramic [VTE]), and customized abutment materials [PEEK and zirconia]). The study agreed that all restorative crown materials and customized abutment materials with different elastic properties had similar biomechanical behavior in terms of stress distribution in implants and peripheral bone, yet it should be noted that this study was also focusing on single implant restorations [33]. 

This was explained by other studies suggesting that despite variations of displacement levels with different restorative materials, the total energy transferred to the implant-bone interface remains similar. Moreover, this energy is transmitted through multiple layers including the crown, cement layer, screw, and abutment, thus absorbed by such intermediate structures [53, 54]. 

Data from the current study may contribute to a valuable basis for restoring posterior edentulous maxilla. It might also provide a better understanding of the biomechanical behavior of different implant distributions and prosthetic materials. The results obtained from this study can also be influenced by other contributing biological and clinical demands.

One limitation of this study is the applied load being static and defined in a fixed axial direction, which did not accurately simulate the cinical dynamic loading, diversity of force angulations and complexity of stresses associated with mastication. Another limitation is that the friction coefficients at the implant and bone interface for all implant configurations are set to be similar and zero. Moreover, the assumptions of homogeneity, isotropicity, linear elasticity, and 100% contact at material interfaces ( prothesis, cement, abutment, implant and bone ) that are standard in FEA studies are ideal assumptions that do not really exist in clinical conditions [28, 55, 56]. 

Considering these points, future FEA studies with different force angulations should be conducted together with in vitro testing of the mechanical behavior of the current prosthetic materials to validate the results and further clinical studies to demonstrate the prognosis of the implant configurations and prosthetic materials in various clinical conditions. Even so, in the light of this study it seems that the combination of implant prosthesis without cantilever, together with a rigid prosthetic material exhibits better biomechanical behavior and stress distribution around bone and implants.

## Conclusions

Within the limitations of this FEA study the following can be concluded:

1. Monolithic zirconia as a rigid prosthetic material transmits less stresses than PEKK to the implant and bone interfaces in implant supported fixed prostheses.
2. Fixed-fixed prosthetic design without cantilever reveals the lowest von Mises stresses with equally distributed stress patterns and most favorable biomechanical behavior.
3. The low elastic modulus PEKK material with distal cantilever design generates the highest stresses to implants and surrounding bone.
4. Mesial cantilever design together with zirconia as a rigid prosthetic material is suggested as a second alternative with acceptable biomechanical behavior in clinically demanding conditions.

## Data Availability

The datasets analysed during the current study available from the corresponding author on reasonable request.

## Declarations

### Regulatory Statement

This research was approved by the committee of Faculty of Dentistry Ain Shams University Research Ethics (FDASU-REC). An informed consent was obtained to participate.
